# Effects of Sodium–Glucose Co-transporter 2 Inhibition with Empaglifozin on Renal Structure and Function in Non-diabetic Rats with Left Ventricular Dysfunction After Myocardial Infarction

**DOI:** 10.1007/s10557-020-06954-6

**Published:** 2020-03-17

**Authors:** Salva R. Yurista, Herman H. W. Silljé, Harry van Goor, Jan-Luuk Hillebrands, Hiddo J. L. Heerspink, Luiz de Menezes Montenegro, Silke U. Oberdorf-Maass, Rudolf A. de Boer, B. Daan Westenbrink

**Affiliations:** 1 Department of Cardiology, University of Groningen, University Medical Center Groningen, Groningen, The Netherlands; 2 Department of Pathology and Medical Biology, Division of Pathology, University of Groningen, University Medical Center Groningen, Groningen, The Netherlands; 3 Department of Clinical Pharmacy and Pharmacology, University of Groningen, University Medical Center Groningen, Groningen, Netherlands

**Keywords:** Sodium–glucose co-transporter 2 inhibitors, Diabetes, Heart failure, Renal function

## Abstract

**Background:**

The use of sodium–glucose co-transporter 2 inhibitors (SGLT2i) is currently expanding to cardiovascular risk reduction in non-diabetic subjects, but renal (side-)effects are less well studied in this setting.

**Methods:**

Male non-diabetic Sprague Dawley rats underwent permanent coronary artery ligation to induce MI, or sham surgery. Rats received chow containing empagliflozin (EMPA) (30 mg/kg/day) or control chow. Renal function and electrolyte balance were measured in metabolic cages. Histological and molecular markers of kidney injury, parameters of phosphate homeostasis and bone resorption were also assessed.

**Results:**

EMPA resulted in a twofold increase in diuresis, without evidence for plasma volume contraction or impediments in renal function in both sham and MI animals. EMPA increased plasma magnesium levels, while the levels of glucose and other major electrolytes were comparable among the groups. Urinary protein excretion was similar in all treatment groups and no histomorphological alterations were identified in the kidney. Accordingly, molecular markers for cellular injury, fibrosis, inflammation and oxidative stress in renal tissue were comparable between groups. EMPA resulted in a slight increase in circulating phosphate and PTH levels without activating FGF23–Klotho axis in the kidney and bone mineral resorption, measured with CTX-1, was not increased.

**Conclusions:**

EMPA exerts profound diuretic effects without compromising renal structure and function or causing significant electrolyte imbalance in a non-diabetic setting. The slight increase in circulating phosphate and PTH after EMPA treatment was not associated with evidence for increased bone mineral resorption suggesting that EMPA does not affect bone health.

**Electronic supplementary material:**

The online version of this article (10.1007/s10557-020-06954-6) contains supplementary material, which is available to authorized users.

## Introduction

Sodium–glucose co-transporter 2 inhibitors (SGLT2i) reduce cardiovascular (CV) events and prevent heart failure (HF) hospitalizations when given to diabetic subjects with either established CV disease or with multiple risk factors for CV disease [1–3]. Furthermore, SGLT2 inhibitors also appear to slow the progression of diabetic kidney disease in patients with type 2 diabetes mellitus (T2D) [4–6]. On the other hand, it has also been suggested that SGLT2 inhibitors promote bone mineral reabsorption and may increase fracture risk in these patients [7–9].

The substantial reduction in HF hospitalizations observed in patients with T2D have led to the hypothesis that these drugs could also be beneficial in non-diabetic patients. Indeed, we and others have recently demonstrated that the SGLT2i EMPA improves cardiac function and ameliorates cardiac remodelling in non-diabetic animals with HF after a large myocardial infarction (MI) [10, 11]. The effects of SGLT2i on clinical outcomes are also currently under investigation in several phase 3 clinical trials that include diabetic and non-diabetic HF patients (NCT03057977, NCT03057951, NCT03036124, NCT03619213). Recently, the Dapagliflozin And Prevention of Adverse-outcomes in Heart Failure (DAPA-HF, NCT03036124) trial revealed that the SGLT2i dapagliflozin reduced the risk of worsening heart failure or cardiovascular mortality, regardless of the presence or absence of diabetes [12]. Importantly, dapagliflozin did not appear to compromise renal function [12].

SGLT2i have potent diuretic properties, which may partially explain their beneficial effects on HF hospitalisations [13, 14]. Nevertheless, the diuretic effects of SGLT2i could also limit the use of SGLT2i in HF patients without T2D. While diuretics are recommended to alleviate congestion in HF patients, their side effects include plasma volume contraction, electrolyte imbalance, renal dysfunction and even kidney injury [15, 16]. As renal dysfunction is strongly associated with impaired outcome in HF patients [16–18], careful titration of diuretics is of paramount importance in HF [16]. While the effects of SGLT2i on renal function are well described in patients with T2D, little is known about the renal effects of SGLT2i in the non-diabetic context. We therefore aimed to determine the effects of empagliflozin (EMPA) on renal structure and function in non-diabetic rats with LV dysfunction after MI, as this is a realistic clinical scenario if not now, likely in the near future.

## Methods

The current analysis represents a renal substudy of a recently published article [10]. We refer to this publication for detailed description of the methods and the cardiometabolic effects.

### Animals

The study was performed in male non-diabetic Sprague Dawley rats weighing 250–280 g (Envigo, The Netherlands). Animals were fed ad libitum and housed conventionally in groups of two to four rats with 12:12 h light–dark cycles. The study was approved by the local Animal Ethics committee (IvD number 16487-02-001) and we followed ARRIVE guidelines when reporting this study [19].

### Myocardial Infarction Surgery

Rats were randomized to HF or sham surgery under isoflurane anaesthesia. HF was induced by permanent ligation of the proximal portion of the left coronary artery as previously described [20, 21]. Sham-operated rats underwent the same procedure but without coronary ligation.

### Investigational Drug

Empagliflozin (BI 10773) was mixed with standard rat chow (R/M-H V1534-70, Ssniff, Germany) in a final concentration of 200 mg/kg intended to reach an average dose of 30 mg/kg/day.

### Experimental Protocol

As described previously, this study represents a renal-oriented substudy of a recently published cohort of non-diabetic rats with LV dysfunction after MI [10]. Rats were treated with EMPA or chow starting either 2 days before surgery (EMPA-early) or 2 weeks after surgery (EMPA-late). After 10 weeks of treatment with EMPA or vehicle, rats were anaesthetized, blood was drawn (either anti-coagulated with EDTA or sodium heparin) and the organs were rapidly excised. Kidney tissues were sectioned transversally and processed for immunohistochemistry or snap-frozen for molecular analysis. Rats with an infarct size of less than 15% were excluded from analysis.

### Metabolic Cage

Two weeks before termination, rats were placed in metabolic cages to monitor 24-h water and food intake and 24-h urine collection, as described [10]. After 24-h, blood samples were drawn from tail vein and plasma was collected. Plasma and urine were stored at − 80 °C for later analysis.

### Histology

The kidney was sectioned transversally, fixed by immersion in 4% buffered formaldehyde solutions for 48 h (Klinipath, The Netherlands) and subsequently embedded in paraffin according to standard procedures. Paraffin sections were stained with periodic acid Schiff (PAS) as described before [22]. Mesangial matrix expansion, focal glomerulosclerosis and interstitial fibrosis were evaluated histomorphologically by an experienced renal pathologist (HvG).

### Blood Measurements

Upon sacrifice, 8 ml of blood was drawn from the abdominal aorta and urine was obtained from the bladder. Routine plasma biochemistries (urea, creatinine, phosphorus, magnesium, calcium, uric acid) were determined by Roche COBAS (Roche Diagnostics, Germany). Plasma parathyroid hormone (PTH) concentration was analysed using Immutopics intact PTH ELISA kit 60-2500 (San Clemente, USA) according to the manufacturer’s protocol. Fibroblast growth factor 23 (FGF-23) level was measured in plasma using LSBio intact FGF-23 ELISA kit according to the manufacturer’s protocol (Seattle, USA). Cross-linked C-terminal telopeptide of type I collagen (CTX-1) concentration in plasma was measured using RatLaps™ EIA kit (IDS, Boldon, UK).

### Urine Measurements

Routine urinalysis (total protein, creatinine and phosphorus) were determined on the Roche COBAS (Roche Diagnostics, Germany). Creatinine clearance, as an estimation for glomerular filtration rate, was calculated from 24 h urinary and plasma creatinine levels as previously described [10, 22].

### Quantitative Real-Time PCR

RNA was extracted from the kidney using TRIzol reagent (Invitrogen Corp., USA) and cDNA was synthesized by QuantiTect Reverse Transcription Kit (Qiagen, The Netherlands) as previously described [10] following manufacturer’s protocol. Relative gene expressions were determined by a qRT-PCR (Bio-Rad Lab, The Netherlands). 36B4 reference gene was used to correct all measured mRNA expression. Primer sequences can be found in the online supplementary Table S1.

### Statistical Analysis

Data are presented as means ± standard errors of the mean (SEM). To compare normally distributed parameters, one-way analysis of variance (ANOVA) followed by Tukey’s post hoc test was used. When data were not normally distributed, a non-parametric Kruskal–Wallis test followed by a Mann–Whitney *U* test with correction for multiple comparisons was used. To compare EMPA and vehicle treatment independent of treatment allocation, an independent *t* test or a Mann–Whitney *U* test was used, where appropriate. Differences were considered significant at *p* < 0.05. IBM SPSS Statistics for Windows, Version 23.0 (IBM Corp, USA) was used to perform all statistical analysis.

## Results

A total of 140 rats were randomized to MI or sham surgery, 47 rats died during the surgical procedure and 20 rats with an infarct < 15% of the left ventricle were excluded from analysis, leaving a total of 73 rats for the current analysis. The final group sizes were 8 for the sham-vehicle (Sham-Veh) group, 19 for sham-EMPA group, 22 for MI-vehicle (MI-Veh) group and 24 for MI-EMPA group.

### General and Cardiac Effects of EMPA

A detailed description of the effects of EMPA on food and fluid intake as well as cardiac structure and function in this cohort have been recently published [10], as a reference they are depicted in Table 1. Food intake was comparable between vehicle and EMPA-treated groups (Table 2), resulting in an average daily intake of 30 mg/kg/day of EMPA. While the size of the MI was comparable between the vehicle and the EMPA treatment groups, EMPA resulted in a marked improved cardiac function and attenuated echocardiographic and histological indices for cardiac remodelling (Table 1).

**Table 1:** Cardiovascular characteristics

Parameters	Sham-Veh	Sham-EMPA	MI-Veh	MI-EMPA
MI size (%)	0	0	31.3 ± 1.5	33.5 ± 2.0
VWs/TL (mg/mm)	30.8 ± 0.4	30.7 ± 0.6	33.9 ± 0.5^#^	30.7 ± 0.6*
LVEF (%)	73 ± 3	75 ± 2	43 ± 2^#^	54 ± 2*
% fibrosis	4.8 ± 1.7	3.95 ± 0.9	19.5 ± 2.5^#^	10.1 ± 0.6*
Cardiomyocyte CSA (μm^2^)	447.9 ± 158.3	450.4 ± 103.3	814.4 ± 49.0^#^	629.1 ± 28.6*
SBP (mmHg)	114 ± 4	116 ± 4	118 ± 4	109 ± 9
DBP (mmHg)	81 ± 4	82 ± 4	85 ± 3	78 ± 8
ANP mRNA (fold change)	1.00 ± 0.18	1.33 ± 0.24	6.51 ± 0.84^#^	3.67 ± 0.61*
β-MHC/α-MHC mRNA ratio (fold change)	1.00 ± 0.18	1.04 ± 0.07	1.98 ± 0.18^#^	1.16 ± 0.07*

Data are presented as means ± SEM

*Veh* vehicle, *EMPA* empagliflozin, *MI* myocardial infarction, *VW/TL* ventricular weight/tibia length, *LVEF* LV ejection fraction, *CSA* cross-sectional area, *SBP* systolic blood pressure, *DBP* diastolic blood pressure, *ANP* atrial natriuretic peptides, *β-MHC* myosin heavy chain isoform beta, *α-MHC* myosin heavy chain isoform alpha

*p < 0.05 vs. MI-Veh; ^#^*p* < 0.05 vs. Sham-Veh

**Table 2:** General characteristics of rats with LV dysfunction and sham-operated animals

Parameters	Sham-Veh	Sham-EMPA	MI-Veh	MI-EMPA
Water intake (ml/24 h)	33.7 ± 0.9	59.8 ± 1.3^#^	31.9 ± 0.6	63.4 ± 1.3*
Food intake (g/24 h)	32.9 ± 0.3	33.3 ± 0.3	32.9 ± 0.3	33.6 ± 0.2
Urine Production (ml/24 h)	13.56 ± 1.16	32.79 ± 1.48^#^	14.35 ± 0.66	34.09 ± 1.40*
Plasma glucose (mmol/l)	13.72 ± 1.47	12.31 ± 0.90	13.72 ± 0.57	12.62 ± 0.87
Plasma sodium (mmol/l)	138.83 ± 1.05	138.88 ± 0.34	139.30 ± 0.36	140.00 ± 0.47
Plasma potassium (mmol/l)	5.08 ± 0.25	4.80 ± 0.12	4.83 ± 0.07	4.79 ± 0.08
Glucose excretion (mmol/day)	0.01 ± 0.01	8.98 ± 0.84^#^	0.01 ± 0.00	11.07 ± 0.92*
Sodium excretion (mmol/day)	1.93 ± 0.08	2.87 ± 0.15^#^	1.85 ± 0.15	3.13 ± 0.17*
Haematocrit (l/l)	45.85 ± 1.39	48.81 ± 0.90	48.42 ± 1.05	49.16 ± 0.71
Insulin/glucagon ratio	4.15 ± 0.77	1.65 ± 0.22^#^	4.42 ± 0.78	1.66 ± 0.10*

Data are presented as means ± SEM

*p < 0.05 vs. MI-Veh; ^#^*p* < 0.05 vs. Sham-Veh

### Effects of EMPA on Renal Structure

**Fig. 1:**
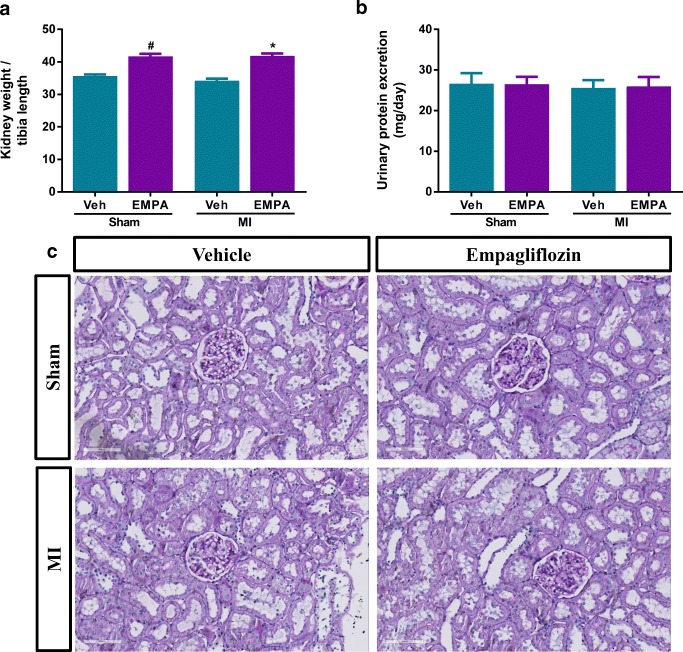
Effect of empagliflozin on parameters of renal structure. **a** Ratio of wet kidney weight to tibia length; *n* = 8–24/group. **b** 24-h urinary protein excretion; *n* = 8–24/group. **c** Representative images of PAS-stained kidney sections (scale bar 100 μm); *n* = 8/group. Veh, vehicle; EMPA, empagliflozin. Data are presented as means ± SEM. *p < 0.05 vs. MI-Veh; ^#^*p* < 0.05 vs. Sham-Veh

To investigate the effect of EMPA on the renal structure, wet weight of the kidney and 24-h protein excretion were measured and kidney sections stained with PAS were analysed. The relative wet kidney weight was slightly increased in sham and MI animals treated with EMPA compared to vehicle-treated rats (Fig. 1a). Daily protein excretion did not differ among the groups, indicating that EMPA did not cause proteinuria (Fig. 1b). Furthermore, histomorphological changes were not observed in sham and MI animals treated with EMPA or vehicle (Fig. 1c).

**Fig. 2:**
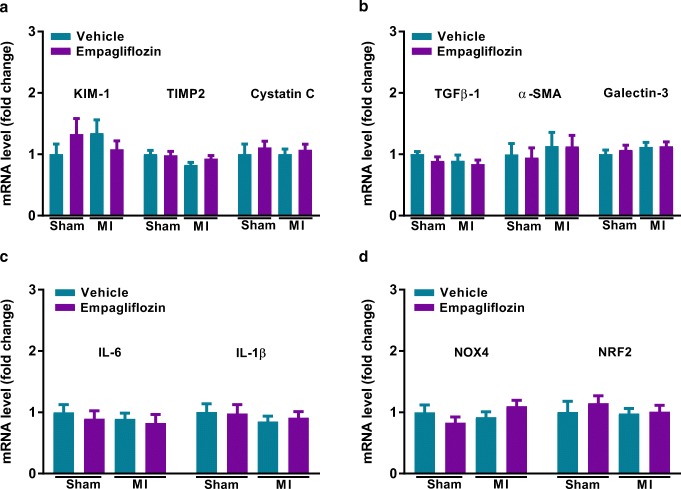
Effect of empagliflozin on markers of kidney damage. **a** Measurements of mRNA levels to assess molecular markers for kidney injury; *n* = 8–24/group. **b** Measurements of mRNA levels to assess molecular markers for fibrosis; *n* = 8–24/group. **c** Measurements of mRNA levels to assess molecular markers for inflammation; *n* = 8–24/group. **d** Measurements of mRNA levels to assess molecular markers for oxidative stress; *n* = 8–24/group. Veh, vehicle; EMPA, empagliflozin. Data are presented as means ± SEM. *p < 0.05 vs. MI-Veh; ^#^*p* < 0.05 vs. Sham-Veh

To assess molecular markers for kidney damage, mRNA expression of markers for kidney injury, fibrosis, inflammation and oxidative stress were determined. The cellular injury markers kidney injury molecule-1 (KIM-1), tissue inhibitor of metalloproteinases 2 (TIMP2) and cystatin C, that is used as a marker to estimate GFR, as well as the kidney fibrosis markers transforming growth factor beta-1 (TGF-β1), alpha-smooth muscle actin (α-SMA) and galectin-3 were comparable between groups (Fig. 2a, b). Moreover, the inflammatory markers interleukin 6 (IL-6) and interleukin 1 beta (IL-1β) and the oxidative stress markers NADPH oxidase 4 (NOX4) and the nuclear factor (erythroid-derived 2)-like 2 (NRF2) were also comparable (Fig. 2c, d). Taken together, our results indicate that the small increase in kidney weight observed in our cohort was not associated with evidence of structural damage to the kidney. The increase in kidney weight is probably caused by non-pathological fluid accumulation that was removed by alcohol solutions in dehydration step.

### Effects of EMPA on Electrolytes and Renal Function

**Fig. 3:**
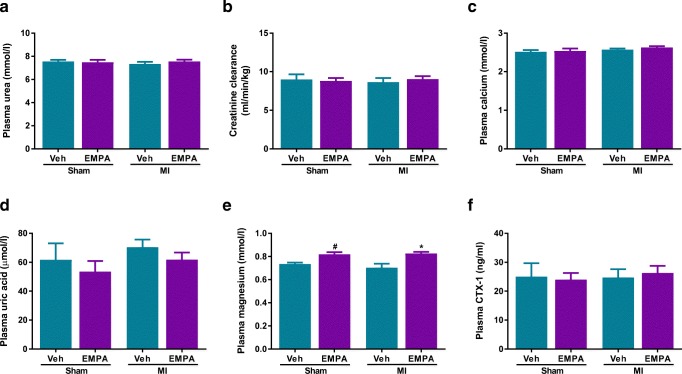
Effect of empagliflozin on renal function, magnesium, calcium and c-terminal telopeptide of type 1 collagen (CTX). **a** Plasma urea; *n* = 8–24/group. **b** Creatinine clearance of all groups; *n* = 8–24/group. **c** Plasma calcium; *n* = 8–24/group. **d** Plasma uric acid; *n* = 8–24/group. **e** Plasma magnesium; *n* = 8–24/group. **f** Plasma C-terminal telopeptide of type I collagen (CTX-1); *n* = 8–12/group. Veh, vehicle; EMPA, empagliflozin. Data are presented as means ± SEM. *p < 0.05 vs. MI-Veh; ^#^*p* < 0.05 vs. Sham-Veh

EMPA resulted an increased in sodium and glucose excretion as well as a twofold increase in urine production (Table 2). Fluid intake was also twofold higher in EMPA-treated sham and MI groups and there was no evidence for plasma volume contraction as evidenced by comparable haematocrit levels (Table 2) and plasma urea concentrations (Fig. 3a). Similarly, creatinine clearance was comparable between the groups (Fig. 3b). No changes were observed in plasma concentrations of glucose, sodium and potassium (Table 2). Plasma calcium and uric acid (Fig. 3c, d) were also comparable between EMPA and vehicle-treated groups. EMPA has been shown to increase magnesium levels in patients with diabetes [23] and we also detected a small increase in plasma magnesium levels in the EMPA-treated sham and MI groups (Fig. 3e).

### Effects of EMPA on Phosphate Homeostasis

Phosphate homeostasis is regulated by a complex process involving intestinal, renal and bone handling [24]. Plasma phosphate plays an essential role on bone health [25]. It has been suggested that SGLT2 inhibitors stimulate diabetes-related bone resorption and can increase the fracture-risk in patients with diabetes [7, 26]. To assess whether this occurs in non-diabetic rats with HF, we evaluated several parameters of phosphate homeostasis namely plasma levels of PTH and FGF23 as well as the renal expression of Klotho and renal type 2a sodium–phosphate co-transporter (NaPi-2a).

**Fig. 4:**
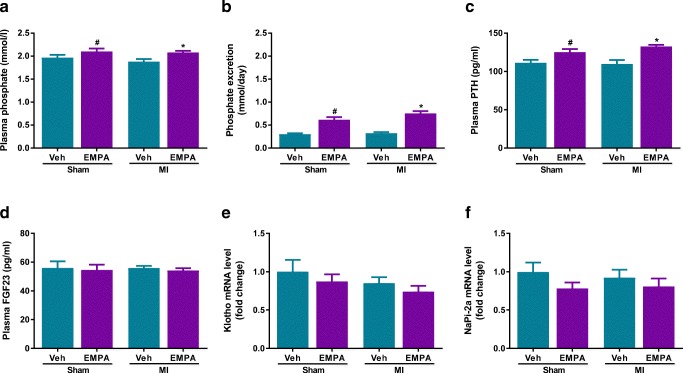
Effect of empagliflozin on phosphate homeostasis. **a** Plasma phosphate; *n* = 8–24/group. **b** Urinary phosphate excretion; *n* = 8–24/group. **c** Plasma parathyroid hormone (PTH); *n* = 8–12/group. **d** Plasma fibroblast growth factor 23 (FGF23); *n* = 8–12/group. **e** mRNA levels of Klotho; *n* = 8–24/group. **f** mRNA levels of type IIa sodium–phosphate co-transporter (NaPi-2a); *n* = 8–24/group. Veh, Vehicle; EMPA, empagliflozin. Data are presented as means ± SEM. *p < 0.05 vs. MI-Veh; ^#^*p* < 0.05 vs. Sham-Veh

EMPA increased plasma phosphate levels by 8% (Fig. 4a) and increased the urinary excretion of phosphate (Fig. 4b). Furthermore, EMPA increased circulating PTH levels (Fig. 4c). Plasma levels of FGF23 were not affected by EMPA (Fig. 4d), nor was kidney expression of Klotho (Fig. 4e) and NaPi-2a (Fig. 4f) different in sham and MI groups. In addition, EMPA had no effect on bone mineral resorption marker CTX-1 in both sham and MI animals. These findings suggest that EMPA marginally increases plasma phosphate and PTH levels without affecting FGF23–Klotho axis thus does not affect bone resorption in a non-diabetic setting.

## Discussion

We previously discovered that the anti-diabetic drug EMPA improves cardiac function in non-diabetic rats with HF after MI, suggesting that it may also benefit non-diabetic HF patients. In the current analysis, we aimed to determine the renal effects of EMPA in non-diabetic HF and were able to demonstrate that (1) the beneficial effects of EMPA on cardiac function and remodelling were not offset by detrimental consequences on renal structure and function. (2) The marked diuretic effects of EMPA were not associated with severe plasma volume contraction, nor did EMPA alter plasma levels of glucose and (most) electrolytes. (3) Structural, functional and molecular analyses did not provide any suggestion for EMPA-induced (long term) renal damage in rats with or without HF. (4) EMPA increased circulating magnesium levels. (5) EMPA increased circulating phosphate and PTH levels without activating the FGF23–Klotho axis. Accordingly, EMPA did not promote bone mineral resorption. These findings suggest that the beneficial effect of EMPA in non-diabetic HF are not offset by renal side effects or an increased fracture risk.

Renal dysfunction is common in HF [27], and worsening renal function (WRF) has been associated with mortality and HF hospitalisations [17]. Moreover, HF has been associated with elevated galectin-3 that play a central role in both heart and kidney fibrosis [28, 29]. Congestion is one of the clinical hallmarks of HF and congestion can also promote renal dysfunction [30, 31]. HF patients are often treated with diuretics to prevent or treat congestion, but little is known about their effect on prognosis [16, 32]. In high doses, diuretics activate the renin–angiotensin–aldosterone system (RAAS) and may thereby promote HF progression [33, 34]. Furthermore, diuretics can cause plasma volume contraction, worsen renal function and can cause various electrolyte disturbances including hypokalemia, hypomagnesemia, hypocalcemia, hyponatremia and hyperuricemia [35–37]. While mineralocorticoid receptor antagonists (MRAs) have mild diuretic effects and do improve prognosis in HF with reduced ejection fraction (EF) [38], hyperkalemia and WRF are common side effects of these drugs as well [39]. Our findings showed that the potent diuretic effects of EMPA are not accompanied by renal dysfunction and electrolyte imbalance are reassuring, and SGLT2i may offer a safe opportunity to alleviate congestion.

An emerging hypothesis is that SGLT2i may directly inhibit the Na+/H+ exchanger isoform 1 (NHE1) in the myocardium and NHE isoform 3 (NHE3) in the kidney [40–43]. It is thought that the suppression of NHE1 by SGLT2i reduces cardiac, injury, hypertrophy and fibrosis as well as reduce in the risk of cardiovascular death and hospitalization for heart failure [40, 41], and AMPK might also mediate indirect SGLT2i–NHE interactions in the heart [44], whereas the suppression of NHE3, which is increased in HF, leads to the inhibition of proximal tubular reabsorption of sodium and thereby decreasing intravascular volume and cardiac wall stress [40, 41, 45]. Thus, inhibition of NHE1 and NHE3 may be beneficial to prevent and/or treat heart failure.

The considerable increase in diuresis observed with SGLT2i suggests that these drugs could be classified as diuretics. A study in patients with T2D demonstrated that SGLT2i dapagliflozin may have a diuretic-like effect beyond the glycaemic control [46]. The mechanisms of SGLT2i-mediated diuresis are, however, very different from classical diuretics prescribed to HF patients. SGLT2i function as osmotic diuretics and reduce interstitial fluid volume without causing major changes to plasma volume and sodium levels, whereas the reverse is true for loop diuretics [47, 48]. Another difference between SGLT2i and classical diuretics is that SGLT2i promotes uric acid excretion and may therefore prevent gout [49]. Nevertheless, the cardiovascular benefits of SGLT2i in high risk exceed the benefits of classical diuretics, making it highly unlikely that these benefits are merely explained by enhanced diuresis [50]. Future studies should better map the interplay and interactions of joint use of thiazides and loop diuretics and SGLT2i.

The US Food and Drug Administration (FDA) has raised the concern for acute kidney injury (AKI) associated with SGLT2i canagliflozin and dapagliflozin use [51]. However, a longitudinal analysis from two large health system registries, Mount Sinai chronic kidney disease registry and the Geisinger Health System cohort, demonstrated that SGLT2i use is not associated with an increased risk of AKI in patients with T2D [52]. Furthermore, a study by Cahn et al. confirmed that SGLT2i do not increase risk for AKI compared with DPP4 inhibitors among patients with T2D [53]. DAPA-HF trial revealed that the beneficial effects of SGLT2i dapagliflozin was not related to any adverse events on renal function [12]. Our findings support and extend this observation as we found no evidence of renal damage, by either functional (plasma urea, creatinine clearance) or structural (proteinuria, renal histopathological analysis) renal damage after EMPA treatment. Similarly, we have performed a comprehensive screening of the relevant markers for kidney injury, fibrosis, inflammation and oxidative stress, and no significant differences were observed among the various groups, providing molecular proof that there are no damaging processes occurring in kidneys of rats treated with SGLT2i.

SGLT2i increases circulating Mg^2+^ levels in individuals with T2D, and therefore, it has been proposed that the increase in circulating Mg^2+^ could at least partially underlie the cardio-beneficial effects of SGLT2i [23, 54, 55]. Mg^2+^ homeostasis is maintained by the interaction of the intestine, bone and kidneys [56, 57]. Several hormones have been suggested to affect the Mg^2+^ balance. Studies in rats showed that Mg^2+^ reabsorptive capacity in the distal segment of the kidney is increased after glucagon and PTH infusion [58, 59]. Insulin causes a shift of magnesium from the extracellular to the intracellular space, resulting in a decrease in plasma magnesium and an increase in erythrocyte magnesium content in both healthy non-diabetics and diabetic individuals [60, 61]. In our study, we observed that EMPA reduced insulin/glucagon ratio (Table 2) and increased PTH levels in plasma (Fig. 4c). Building upon these observations, our data suggest that increases in plasma magnesium levels are most probably explained by the effects of EMPA on insulin, glucagon and PTH.

Our observation demonstrated that EMPA caused a slight but significant increase in serum phosphate and PTH concentrations and is in line with similar observations in patients with or without diabetes [62–64]. The increase in PTH levels has raised the concern that SGLT2i may promote bone mineral resorption and increase fracture risk [62]. The putative mechanisms are sought in the fact that SGLT2i increase tubular sodium concentrations and increase renal phosphate reabsorption through enhanced activity of sodium–phosphate co-transporter (NaPi) [62]. The resultant increase in plasma phosphate levels will then trigger a reactive increase in PTH secretion by the parathyroid gland and the secretion of FGF23 by osteocytes. PTH and FGF-23 promote bone resorption and reduce bone mineralisation, respectively, and are both implicated in osteoporosis [24, 65]. Furthermore, both factors suppress the activity of NaPi-2a and NaPi-2c in the kidney which promotes urinary phosphate excretion [66]. A study by Weir et al. demonstrated that in patients with T2D receiving SGLT2i canagliflozin versus placebo, minimal increases in serum phosphate and magnesium were within normal limits and has no clinical relevance [67]. While we did observe an increase in plasma phosphate and PTH levels, the observed changes were within physiological range [68]. Furthermore, calcium levels were unaltered and we did not find evidence for enhanced bone resorption. Plasma FGF23 and renal expression of NaPi-2a were also unaltered, indicating that EMPA did not result in the activation of the FGF-23–Klotho axis. In addition, no effect on plasma CTX-1 was noted in both vehicle and EMPA-treated animals. These results are in contrast with observations by Thraikill et al., who studied a model for diabetic bone disease [7]. Our findings are also in line with recent evidence that demonstrated that SGLT2i do not result in a net increase in fracture risk [69, 70]. Taken together, our data suggest that EMPA does not affect the bone mineral resorption in a non-diabetic context.

## Study Limitations

Despite strengths related to the direct measures of renal structure, function and other physiological parameters, our study does have limitations. In addition to the inherent limitations associated with our experimental heart failure model and differences among species, there are a number of specific limitations to our study. First, in accordance with the previous studies, our post-MI HF model did not induce renal dysfunction in rats [71, 72]. Our findings may therefore not reflect the effects of EMPA in patients with both cardiac and renal dysfunction. Second, we compared EMPA with placebo in this study and therefore cannot extrapolate whether there will be a meaningful interaction with other HF drugs, including diuretics. Third, our study was not focussed on bone health and we did not perform direct measurements of bone mineral density. Nevertheless, our study does provide compelling evidence suggesting that the cardiac benefits of EMPA are not associated with detrimental renal consequences or impediments in bone health. Another potential limitation of our study was the exclusion of 20 rats with small infarctions because their cardiac function is close to normal. As a sensitivity analysis we performed all analyses both with and without rats with small infarctions, and no differences in the outcomes were observed (data not shown). Further research is clearly required to ascertain whether the improvement of cardiac function was associated with diuretic properties of EMPA treatment or improvement of cardiac function caused the diuresis.

## Conclusion

SGLT2 inhibition with EMPA exerts profound diuretic effects without compromising renal structure and function or causing significant electrolyte imbalance. The slight increase in circulating phosphate and PTH after EMPA treatment was not associated with evidence for increased bone mineral resorption suggesting that EMPA does not affect bone health.

## Electronic Supplementary Material

ESM 1 (PDF 152 kb)

## Abbreviations

SGLT2: Sodium–glucose co-transporter 2
SGLT2i: Sodium–glucose co-transporter 2 inhibitors
CV: Cardiovascular
HF: Heart failure
T2D: Type 2 diabetes mellitus
EMPA: Empagliflozin
LV: Left ventricular
MI: Myocardial infarction
PTH: Parathyroid hormone
FGF-23: Fibroblast growth factor 23
CTX-1: Cross-linked C-terminal telopeptide of type I collagen
KIM-1: Kidney injury molecule-1
TIMP2: Tissue inhibitor of metalloproteinases 2
TGF-β1: Transforming growth factor beta-1
α-SMA: Alpha-smooth muscle actin
IL-6: Interleukin 6
IL-1β: Interleukin 1 beta
NOX4: NADPH oxidase 4
NRF2: Nuclear factor (erythroid-derived 2)-like 2
RAAS: Renin–angiotensin–aldosterone system
MRAs: Mineralocorticoid receptor antagonists
EF: Ejection fraction
AKI: Acute kidney injury
NaPi: Sodium–phosphate cotransporter

## References

[1] Zinman B, Wanner C, Lachin JM, Fitchett D, Bluhmki E, Hantel S, Mattheus M, Devins T, Johansen OE, Woerle HJ, Broedl UC, Inzucchi SE, EMPA-REG OUTCOME Investigators (2015). Empagliflozin, cardiovascular outcomes, and mortality in type 2 diabetes. N Engl J Med.

[2] Neal B, Perkovic V, Mahaffey KW, de Zeeuw D, Fulcher G, Erondu N, Shaw W, Law G, Desai M, Matthews DR, CANVAS Program Collaborative Group (2017). Canagliflozin and cardiovascular and renal events in type 2 diabetes. N Engl J Med.

[3] Wiviott Stephen D., Raz Itamar, Bonaca Marc P., Mosenzon Ofri, Kato Eri T., Cahn Avivit, Silverman Michael G., Zelniker Thomas A., Kuder Julia F., Murphy Sabina A., Bhatt Deepak L., Leiter Lawrence A., McGuire Darren K., Wilding John P.H., Ruff Christian T., Gause-Nilsson Ingrid A.M., Fredriksson Martin, Johansson Peter A., Langkilde Anna-Maria, Sabatine Marc S. (2019). Dapagliflozin and Cardiovascular Outcomes in Type 2 Diabetes. New England Journal of Medicine.

[4] Wanner C, Inzucchi SE, Lachin JM, Fitchett D, von Eynatten M, Mattheus M, Johansen OE, Woerle HJ, Broedl UC, Zinman B, EMPA-REG OUTCOME Investigators (2016). Empagliflozin and progression of kidney disease in type 2 diabetes. N Engl J Med.

[5] Perkovic V, de Zeeuw D, Mahaffey KW, Fulcher G, Erondu N, Shaw W (2018). Canagliflozin and renal outcomes in type 2 diabetes: results from the CANVAS Program randomised clinical trials. Lancet Diabetes Endocrinol.

[6] Mosenzon O, Wiviott SD, Cahn A, Rozenberg A, Yanuv I, Goodrich EL, Murphy SA, Heerspink HJL, Zelniker TA, Dwyer JP, Bhatt DL, Leiter LA, McGuire DK, Wilding JPH, Kato ET, Gause-Nilsson IAM, Fredriksson M, Johansson PA, Langkilde AM, Sabatine MS, Raz I (2019). Effects of dapagliflozin on development and progression of kidney disease in patients with type 2 diabetes: an analysis from the DECLARE–TIMI 58 randomised trial. Lancet Diabetes Endocrinol.

[7] Thrailkill KM, Clay Bunn R, Nyman JS, Rettiganti MR, Cockrell GE, Wahl EC (2016). SGLT2 inhibitor therapy improves blood glucose but does not prevent diabetic bone disease in diabetic DBA/2J male mice. Bone..

[8] de Jong MA, Petrykiv SI, Laverman GD, van Herwaarden AE, de Zeeuw D, Bakker SJL (2019). Effects of dapagliflozin on circulating markers of phosphate homeostasis. Clin J Am Soc Nephrol.

[9] Kohan DE, Fioretto P, Tang W, List JF (2014). Long-term study of patients with type 2 diabetes and moderate renal impairment shows that dapagliflozin reduces weight and blood pressure but does not improve glycemic control. Kidney Int.

[10] Yurista SR, Silljé HHW, Oberdorf-Maass SU, Schouten E, Pavez Giani MG, Hillebrands J, van Goor H, van Veldhuisen D, de Boer RA, Westenbrink BD (2019). Sodium-glucose co-transporter 2 inhibition with empagliflozin improves cardiac function in non-diabetic rats with left ventricular dysfunction after myocardial infarction. Eur J Heart Fail.

[11] Santos-Gallego CG, Requena-Ibanez JA, San Antonio R, Ishikawa K, Watanabe S, Picatoste B (2019). Empagliflozin ameliorates adverse left ventricular remodeling in nondiabetic heart failure by enhancing myocardial energetics. J Am Coll Cardiol.

[12] McMurray JJ V, Solomon SD, Inzucchi SE, Køber L, Kosiborod MN, Martinez FA, et al. Dapagliflozin in Patients with Heart Failure and Reduced Ejection Fraction. N Engl J Med. 2019;381:1995–2008. 10.1056/NEJMoa1911303 31535829

[13] Flores E, Santos-Gallego CG, Diaz-Mejía N, Badimon JJ (2018). Do the SGLT-2 inhibitors offer more than hypoglycemic activity?. Cardiovasc Drugs Ther.

[14] Gupte M, Umbarkar P, Lal H (2017). Mechanistic insights of empagliflozin-mediated cardiac benefits: nearing the starting line. Cardiovasc Drugs Ther.

[15] van Veldhuisen DJ, Ruilope LM, Maisel AS, Damman K (2016). Biomarkers of renal injury and function: diagnostic, prognostic and therapeutic implications in heart failure. Eur Heart J.

[16] Mullens W, Damman K, Harjola V-P, Mebazaa A, Brunner-La Rocca H-P, Martens P (2019). The use of diuretics in heart failure with congestion—a position statement from the Heart Failure Association of the European Society of Cardiology. Eur J Heart Fail.

[17] Damman K, Testani JM (2015). The kidney in heart failure: an update. Eur Heart J.

[18] Urso C, Brucculeri S, Caimi G (2015). Acid–base and electrolyte abnormalities in heart failure: pathophysiology and implications. Heart Fail Rev.

[19] Kilkenny C, Browne WJ, Cuthill IC, Emerson M, Altman DG (2010). Improving bioscience research reporting: the ARRIVE guidelines for reporting animal research. PLoS Biol.

[20] Yin M, van der Horst ICC, van Melle JP, Qian C, van Gilst WH, Silljé HHW (2011). Metformin improves cardiac function in a nondiabetic rat model of post-MI heart failure. Am J Physiol Heart Circ Physiol.

[21] Booij HG, Yu H, De Boer RA, van de Kolk CWA, van de Sluis B, Van Deursen JM (2016). Overexpression of A kinase interacting protein 1 attenuates myocardial ischaemia/reperfusion injury but does not influence heart failure development. Cardiovasc Res.

[22] Frenay A-RS YL, van der Velde AR, Vreeswijk-Baudoin I, López-Andrés N, van Goor H (2015). Pharmacological inhibition of galectin-3 protects against hypertensive nephropathy. Am J Physiol Physiol.

[23] Tang H, Zhang X, Zhang J, Li Y, Del Gobbo LC, Zhai S (2016). Elevated serum magnesium associated with SGLT2 inhibitor use in type 2 diabetes patients: a meta-analysis of randomised controlled trials. Diabetologia..

[24] Blaine J, Chonchol M, Levi M (2015). Renal control of calcium, phosphate, and magnesium homeostasis. Clin J Am Soc Nephrol.

[25] Bonjour J-P (2011). Calcium and phosphate: a duet of ions playing for bone health. J Am Coll Nutr.

[26] Watts NB, Bilezikian JP, Usiskin K, Edwards R, Desai M, Law G, Meininger G (2016). Effects of canagliflozin on fracture risk in patients with type 2 diabetes mellitus. J Clin Endocrinol Metab.

[27] Metra M, Cotter G, Gheorghiade M, Dei Cas L, Voors AA (2012). The role of the kidney in heart failure. Eur Heart J.

[28] Melenovsky V, Cervenka L, Viklicky O, Franekova J, Havlenova T, Behounek M, Chmel M, Petrak J (2018). Kidney response to heart failure: proteomic analysis of cardiorenal syndrome. Kidney Blood Press Res.

[29] Hundae A, McCullough PA (2014). Cardiac and renal fibrosis in chronic cardiorenal syndromes. Nephron Clin Pract.

[30] Sinkeler SJ, Damman K, van Veldhuisen DJ, Hillege H, Navis G (2012). A re-appraisal of volume status and renal function impairment in chronic heart failure: combined effects of pre-renal failure and venous congestion on renal function. Heart Fail Rev.

[31] Pellicori P, Cleland JGF, Zhang J, Kallvikbacka-Bennett A, Urbinati A, Shah P, Kazmi S, Clark AL (2016). Cardiac dysfunction, congestion and loop diuretics: their relationship to prognosis in heart failure. Cardiovasc Drugs Ther.

[32] Ponikowski Piotr, Voors Adriaan A., Anker Stefan D., Bueno Héctor, Cleland John G. F., Coats Andrew J. S., Falk Volkmar, González-Juanatey José Ramón, Harjola Veli-Pekka, Jankowska Ewa A., Jessup Mariell, Linde Cecilia, Nihoyannopoulos Petros, Parissis John T., Pieske Burkert, Riley Jillian P., Rosano Giuseppe M. C., Ruilope Luis M., Ruschitzka Frank, Rutten Frans H., van der Meer Peter (2016). 2016 ESC Guidelines for the diagnosis and treatment of acute and chronic heart failure. European Heart Journal.

[33] Francis GS, Benedict C, Johnstone DE, Kirlin PC, Nicklas J, Liang CS (1990). Comparison of neuroendocrine activation in patients with left ventricular dysfunction with and without congestive heart failure. A substudy of the Studies of Left Ventricular Dysfunction (SOLVD). Circulation..

[34] Ozierański K, Balsam P, Kapłon-Cieślicka A, Tymińska A, Kowalik R, Grabowski M, Peller M, Wancerz A, Marchel M, Crespo-Leiro MG, Maggioni AP, Drożdż J, Filipiak KJ, Opolski G (2019). Comparative analysis of long-term outcomes of torasemide and furosemide in heart failure patients in heart failure registries of the European Society of Cardiology. Cardiovasc Drugs Ther.

[35] Gottlieb SS, Brater DC, Thomas I, Havranek E, Bourge R, Goldman S, Dyer F, Gomez M, Bennett D, Ticho B, Beckman E, Abraham WT (2002). BG9719 (CVT-124), an A1 adenosine receptor antagonist, protects against the decline in renal function observed with diuretic therapy. Circulation..

[36] Oh SW, Han SY (2015). Loop diuretics in clinical practice. Electrolytes Blood Press.

[37] Tamargo J, Caballero R, Delpón E (2018). New therapeutic approaches for the treatment of hyperkalemia in patients treated with renin-angiotensin-aldosterone system inhibitors. Cardiovasc Drugs Ther.

[38] Zannad F, Gattis Stough W, Rossignol P, Bauersachs J, McMurray JJV, Swedberg K (2012). Mineralocorticoid receptor antagonists for heart failure with reduced ejection fraction: integrating evidence into clinical practice. Eur Heart J.

[39] Cooper LB, Lippmann SJ, Greiner MA, Sharma A, Kelly JP, Fonarow GC, et al. Use of Mineralocorticoid Receptor Antagonists in Patients With Heart Failure and Comorbid Diabetes Mellitus or Chronic Kidney Disease. J Am Heart Assoc. 2017;6. 10.1161/JAHA.117.006540. 10.1161/JAHA.117.006540 PMC5779000 29275368

[40] Packer M (2017). Activation and inhibition of sodium-hydrogen exchanger is a mechanism that links the pathophysiology and treatment of diabetes mellitus with that of heart failure. Circulation..

[41] McCullough PA, Kluger AY, Tecson KM, Barbin CM, Lee AY, Lerma EV, Rosol ZP, Kluger SL, Rangaswami J (2018). Inhibition of the sodium-proton antiporter (exchanger) is a plausible mechanism of potential benefit and harm for drugs designed to block sodium glucose co-transporter 2. Rev Cardiovasc Med.

[42] Kluger AY, Tecson KM, Barbin CM, Lee AY, Lerma EV, Rosol ZP (2018). Cardiorenal outcomes in the CANVAS, DECLARE-TIMI 58, and EMPA-REG OUTCOME trials: a systematic review. Rev Cardiovasc Med.

[43] Iborra-Egea O, Santiago-Vacas E, Yurista SR, Lupón J, Packer M, Heymans S (2019). Unraveling the molecular mechanism of action of empagliflozin in heart failure with reduced ejection fraction with or without diabetes. JACC Basic Transl Sci.

[44] Ye Y, Jia X, Bajaj M, Birnbaum Y (2018). Dapagliflozin attenuates Na+/H+ exchanger-1 in cardiofibroblasts via AMPK activation. Cardiovasc Drugs Ther.

[45] Gallo LA, Wright EM, Vallon V (2015). Probing SGLT2 as a therapeutic target for diabetes: basic physiology and consequences. Diab Vasc Dis Res.

[46] Lambers Heerspink HJ, de Zeeuw D, Wie L, Leslie B, List J (2013). Dapagliflozin a glucose-regulating drug with diuretic properties in subjects with type 2 diabetes. Diabetes Obes Metab.

[47] Sattar N, McLaren J, Kristensen SL, Preiss D, McMurray JJ (2016). SGLT2 inhibition and cardiovascular events: why did EMPA-REG outcomes surprise and what were the likely mechanisms?. Diabetologia..

[48] Hallow KM, Helmlinger G, Greasley PJ, McMurray JJV, Boulton DW (2018). Why do SGLT2 inhibitors reduce heart failure hospitalization? A differential volume regulation hypothesis. Diabetes Obes Metab.

[49] Wilcox CS, Shen W, Boulton DW, Leslie BR, Griffen SC. Interaction Between the Sodium‐Glucose–Linked Transporter 2 Inhibitor Dapagliflozin and the Loop Diuretic Bumetanide in Normal Human Subjects. J Am Heart Assoc. 2018;7. 10.1161/JAHA.117.007046. 10.1161/JAHA.117.007046 PMC5850181 29440005

[50] Scheen AJ (2016). Reappraisal of the diuretic effect of empagliflozin in the EMPA-REG OUTCOME trial: comparison with classic diuretics. Diabetes Metab.

[51] US Food and Drug Administration. FDA Drug Safety Communication: FDA strengthens kidney warnings for diabetes medicines canagliflozin (Invokana, Invokamet) and dapagliflozin (Farxiga, Xigduo XR) [Internet]. 2016 [cited 2019 Aug 29]. Available from: https://www.fda.gov/Drugs/DrugSafety/ucm505860.htm.

[52] Nadkarni GN, Ferrandino R, Chang A, Surapaneni A, Chauhan K, Poojary P, Saha A, Ferket B, Grams ME, Coca SG (2017). Acute kidney injury in patients on SGLT2 inhibitors: a propensity-matched analysis. Diabetes Care.

[53] Cahn A, Melzer-Cohen C, Pollack R, Chodick G, Shalev V (2019). Acute renal outcomes with sodium-glucose co-transporter-2 inhibitors: real-world data analysis. Diabetes Obes Metab.

[54] Gilbert RE, Mende C, Vijapurkar U, Sha S, Davies MJ, Desai M (2017). Effects of canagliflozin on serum magnesium in patients with type 2 diabetes mellitus: a post hoc analysis of randomized controlled trials. Diabetes Ther.

[55] Filippatos TD, Tsimihodimos V, Liamis G, Elisaf MS (2018). SGLT2 inhibitors-induced electrolyte abnormalities: an analysis of the associated mechanisms. Diabetes Metab Syndr.

[56] Jahnen-Dechent W, Ketteler M (2012). Magnesium basics. Clin Kidney J.

[57] Gommers LMM, Hoenderop JGJ, Bindels RJM, de Baaij JHF (2016). Hypomagnesemia in type 2 diabetes: a vicious circle?. Diabetes..

[58] Bailly C, Roinel N, Amiel C (1985). Stimulation by glucagon and PTH of Ca and Mg reabsorption in the superficial distal tubule of the rat kidney. Pflugers Arch.

[59] Quamme GA (1997). Renal magnesium handling: new insights in understanding old problems. Kidney Int.

[60] Xu Li Hao Richie, Maalouf Naim M. (2016). Effect of acute hyperinsulinemia on magnesium homeostasis in humans. Diabetes/Metabolism Research and Reviews.

[61] Paolisso G, Sgambato S, Passariello N, Giugliano D, Scheen A, D’Onofrio F (1986). Insulin induces opposite changes in plasma and erythrocyte magnesium concentrations in normal man. Diabetologia..

[62] Taylor SI, Blau JE, Rother KI (2015). Possible adverse effects of SGLT2 inhibitors on bone. Lancet Diabetes Endocrinol.

[63] Filippatos TD, Tsimihodimos V, Liamis G, Elisaf MS (2018). SGLT2 inhibitors-induced electrolyte abnormalities: an analysis of the associated mechanisms. Diabetes Metab Syndr Clin Res Rev.

[64] Blau JE, Bauman V, Conway EM, Piaggi P, Walter MF, Wright EC, et al. Canagliflozin triggers the FGF23/1,25-dihydroxyvitamin D/PTH axis in healthy volunteers in a randomized crossover study. JCI insight. 2018;3. 10.1172/jci.insight.99123. 10.1172/jci.insight.99123 PMC5931122 29669938

[65] Murali SK, Roschger P, Zeitz U, Klaushofer K, Andrukhova O, Erben RG (2016). FGF23 regulates bone mineralization in a 1,25(OH) 2 D 3 and Klotho-independent manner. J Bone Miner Res.

[66] Shimada T, Hasegawa H, Yamazaki Y, Muto T, Hino R, Takeuchi Y (2003). FGF-23 is a potent regulator of vitamin D metabolism and phosphate homeostasis. J Bone Miner Res.

[67] Weir MR, Kline I, Xie J, Edwards R, Usiskin K (2014). Effect of canagliflozin on serum electrolytes in patients with type 2 diabetes in relation to estimated glomerular filtration rate (eGFR). Curr Med Res Opin.

[68] Kohn DF, Clifford CB. Biology and diseases of rats. Lab Anim Med. Elsevier. 2002:121–65.

[69] Tang HL, Li DD, Zhang JJ, Hsu YH, Wang TS, Zhai SD, Song YQ (2016). Lack of evidence for a harmful effect of sodium-glucose co-transporter 2 (SGLT2) inhibitors on fracture risk among type 2 diabetes patients: a network and cumulative meta-analysis of randomized controlled trials. Diabetes Obes Metab.

[70] Abrahami Devin, Douros Antonios, Yin Hui, Yu Oriana H.Y., Azoulay Laurent (2019). Sodium–Glucose Cotransporter 2 Inhibitors and the Risk of Fractures Among Patients With Type 2 Diabetes. Diabetes Care.

[71] Windt WAKM, Henning RH, Kluppel ACA, Xu Y, de Zeeuw D, van Dokkum RPE (2008). Myocardial infarction does not further impair renal damage in 5/6 nephrectomized rats. Nephrol Dial Transplant.

[72] Bongartz LG, Braam B, Gaillard CA, Cramer MJ, Goldschmeding R, Verhaar MC (2012). Target organ cross talk in cardiorenal syndrome: animal models. Am J Physiol Physiol.

