# Supplementary material for: Effects of Sodium–Glucose Co-transporter 2 Inhibition with Empaglifozin on Renal Structure and Function in Non-diabetic Rats with Left Ventricular Dysfunction After Myocardial Infarction
Source: Cardiovasc Drugs Ther. 2020 Mar 17;34(3):311–21. doi: 10.1007/s10557-020-06954-6 (PMC7242237; doi:10.1007/s10557-020-06954-6)
Supplement: Supplementary file 1 — (PDF 152 kb) [file 10557_2020_6954_MOESM1_ESM.pdf]

Supplementary material

Effects of sodium–glucose co-transporter 2 inhibition with empaglifozin on renal structure and function in non-diabetic rats with left ventricular dysfunction after myocardial infarction

Salva R. Yurista MD[1], Herman H.W. Silljé PhD[1], Harry van Goor PhD[2], Jan-Luuk Hillebrands PhD[2], Hiddo J.L. Heerspink PhD[3], Luiz de Menezes Montenegro[1], Silke U. Oberdorf-Maass[1], Rudolf A. de Boer MD PhD[1], B. Daan Westenbrink, MD PhD[1]

[1]Department of Cardiology, University Medical Center Groningen, University of Groningen, Groningen, The Netherlands

[2]Department of Pathology and Medical Biology, Division of Pathology, University Medical Center Groningen, University of Groningen, Groningen, The Netherlands

[3]Department of Clinical Pharmacy and Pharmacology, University of Groningen, University Medical Center Groningen, Groningen, Netherlands

Corresponding author:

B. Daan Westenbrink MD PhD

Department of Cardiology,

University Medical Center Groningen

PO Box 30.001

9700 RB, Groningen, the Netherlands

Phone : +31 50 361 2355 / Fax: +31 50 361 4391 / E-mail: b.d.westenbrink@umcg.nl

Supplementary table 1. Sequnces of primers used for quantitative RT-PCR analysis

Genes	Forward primer (5'-3')	Reverse primer (5'-3')
**KIM-1**	AGAGAGAGCAGGACACAGGCTT	ACCCGTGGTAGTCCCAAACA
**TIMP2**	TGGACGTTGGAGGAAAGAAG	TGTCCCAGGGCACAATAAAG
**Cystatin C**	AGCGAGTACAACAAGGGCAGCAAC	TTGTCAGGGTGTGTGTGCCTTTCC
**TGF-β1**	AAGAAGTCACCCGCGTGCTA	TGTGTGATGTCTTTGGTTTTGTCA
**α-SMA**	CATCATGCGTCTGGACTTGG	TCACGCTCAGCAGTAGTCAC
**Galectin-3**	CCCGCTTCAATGAGAACAAC	ACCGCAACCTTGAAGTGGTC
**IL-6**	AGCGATGATGCACTGTCAGA	TAGCACACTAGGTTTGCCGA
**IL-1β**	TGTGATGAAAGACGGCACACC	GGGAACTGTGCAGACTCAAC
**NOX4**	ATGTTGGGCCTAGGATTGTGT	AAGTTCAGGGCGTTCACCAA
**NRF2**	ATCCAGACAGACACCAGTGGATC	GGCAGTGAAGACTGAACTTTCA
**36B4**	GTTGCCTCAGTGCCTCACTC	GCAGCCGCAAATGCAGATGG
**Klotho**	CGTGAATGAGGCTCTGAAAGC	GAGCGGTCACTAAGCGAATACG
**NaPi-IIa**	CCAACATCGGGACCTCTGTC	CCACCACGGATGTTGAAGGA
**ANP**	ATGGGCTCCTTCTCCATCAC	TCTACCGGCATCTTCTCCTC
**α-MHC**	GACAACTCCTCCCGCTTTGG	AAGATCACCCGGGACTTCTC
**β-MHC**	TCTGGAGGCCTTTGGCAATG	GATGCCAACTTTCCTGTTGC
